# Case Report: When cardiomyopathy is the clue: late-presenting propionic acidemia manifesting as acute heart failure

**DOI:** 10.3389/fped.2026.1891137

**Published:** 2026-10-05

**Authors:** Belinda Kwartemaa Nti, Noah Trapp, Asma Ali, Brendan Lanpher, Jeffrey Weatherhead

**Affiliations:** 1 Department of Pediatrics and Adolescent Medicine, Mayo Clinic, Rochester, MN, United States; 2 Department of Clinical Genomics, Mayo Clinic, Rochester, MN, United States; 3 Department of Pediatrics and Adolescent Medicine, Division of Pediatric Critical Care Medicine, Mayo Clinic, Rochester, MN, United States

**Keywords:** Amish population, dilated cardiomyopathy, heart failure, inborn errors of metabolism, propionic acidemia

## Abstract

**Background:**

Propionic acidemia (PA) is a rare inborn error of metabolism that typically presents in infancy; however, late-onset variants may manifest with atypical features, including cardiac involvement. Dilated cardiomyopathy (DCM) is an uncommon but serious complication.

**Case summary:**

A 15-year-old with no prior medical history but a significant maternal family history of cardiomyopathy of unknown etiology from an Amish background presented with altered mental status, increased work of breathing, and progressive edema in the context of preceding viral illness. Evaluation revealed severe biventricular dysfunction, chamber dilation, and findings consistent with chronic DCM. Despite supportive management, her course was complicated by ventricular arrhythmias and clinical deterioration. Metabolic workup demonstrated elevated propionylcarnitine and increased urinary 2-methylcitric acid, confirming late-onset variant PA. She was treated with intravenous carnitine and a specialized metabolic formula; however, cardiac function remained severely impaired. In accordance with family wishes, she was discharged home on hospice care.

**Conclusion:**

Late-onset PA can present as isolated cardiomyopathy in adolescence. Early consideration of metabolic etiologies is essential in unexplained DCM, particularly in high-risk populations.

## Introduction

Organic acidemias make up a varied group of inborn errors of metabolism that are identified by the excessive accumulation of organic acids, developing into multisystemic disease manifestations. They are among the uncommon causes of dilated cardiomyopathy (DCM) (1, 2). Within the groups of organic acidemias, propionic acidemia (PA) is most associated with cardiac involvement (2).

Clinically, PA usually presents during the neonatal period with vomiting, poor feeding, metabolic acidosis, hyperammonemia, and lethargy. However, late-onset or variant forms may appear after infancy with more subtle or intermittent symptoms, often triggered by infection, fasting, or other metabolic stressors (3). Cardiac manifestations—including dilated or hypertrophic cardiomyopathy, prolonged QT interval, and heart failure—have been increasingly recognized as important complications contributing to morbidity and mortality in these patients (2). We describe the case of a 15-year-old female with previously undiagnosed PA presenting with acute decompensated heart failure secondary to dilated cardiomyopathy. This report highlights the importance of considering metabolic disorders in adolescents presenting with unexplained cardiomyopathy, particularly in populations with a higher prevalence of inborn errors of metabolism. The Amish of North America have an increased prevalence of PA due to a founder hypomorphic missense variant in *PCCB*. The Amish PA phenotype is variable, and some individuals remain asymptomatic and undiagnosed until adulthood.

## Case presentation

A 15-year-old female of Amish background with no previously known medical conditions presented acutely at an outside facility after developing altered mental status while accompanying her grandmother to a clinic appointment. During the initial evaluation, she was placed on cardiac monitoring, which revealed findings concerning for SVT, prompting transfer to the nearest emergency department.

Further history revealed she had been experiencing a 3-week history of flu-like symptoms, followed by progressive abdominal fullness and diarrhea over the preceding week. Four days prior to presentation, she developed worsening bilateral lower extremity edema, increased work of breathing, and decreased oral intake. There was no history of altered mental status prior to presentation, no known sick contacts, and no family history of congenital heart disease. On arrival at the emergency department, she appeared cachectic, tachypneic, tachycardic, and in respiratory distress, answering questions in short sentences. Physical examination revealed bilateral pitting edema (2+) and diminished air entry on chest auscultation bilaterally. No abdominal masses were palpable.

Electrocardiogram showed sinus tachycardia with nonspecific T-wave inversions, most prominent in the anterior and lateral leads, and a QTc of 516 ms. Point-of-care ultrasound revealed a severely reduced ejection fraction with marked atrial and ventricular dilation, consistent with dilated cardiomyopathy. No pericardial effusion was noted. Laboratory workup showed elevated liver enzymes and bilirubin suggestive of hepatic congestion, elevated BNP, and mild lymphocytosis. A CT of the chest and abdomen with contrast showed no evidence of pulmonary embolism; however, it confirmed findings consistent with congestive hepatopathy.

She was placed on BiPAP for respiratory support, started empirically on ceftriaxone and doxycycline, and subsequently transferred to our Intensive Care Unit (ICU) for further management. Additional history upon arrival revealed an extensive positive history of cardiomyopathy due to PA on the maternal side. She had experienced slower growth compared to peers and had repeated the fourth grade, although her parents did not consider her to have significant learning difficulties. Newborn screening was not performed for her. Based on family history, none of the patient's siblings had undergone newborn screening; therefore, this was likely declined rather than unavailable, although this cannot be confirmed. She remained tachypneic and tachycardic with ongoing respiratory distress upon arrival. Milrinone infusion was initiated, and initial laboratory evaluation revealed a pH of 7.39, pCO_2_ of 30.5 mmHg, pO_2_ of 39 mmHg, base excess of −7, anion gap of 22, ionized calcium of 4.5 mg/dL, and lactate of 1.51 mmol/L. She received intravenous calcium gluconate, furosemide, and thiamine. Given the clinical presentation, differentials included viral myocarditis, Lyme disease, nutritional or inflammatory cardiomyopathy, and inborn errors of metabolism. Considering her Amish background and the higher prevalence of organic acidemias in this population—particularly propionic acidemia—a comprehensive metabolic evaluation was performed.

Formal echocardiography revealed biventricular and left atrial enlargement, severely reduced biventricular systolic function, severe mitral regurgitation, and an ejection fraction of 14%, findings most consistent with chronic dilated cardiomyopathy (Supplementary Table S6; Figures 1, 2). She had high anion gap acidosis with normal lactate. Nutritional assessment demonstrated severe malnutrition (MUAC *Z*-score −4), placing her at high risk for refeeding syndrome, which was carefully monitored.

**Figure 1:**
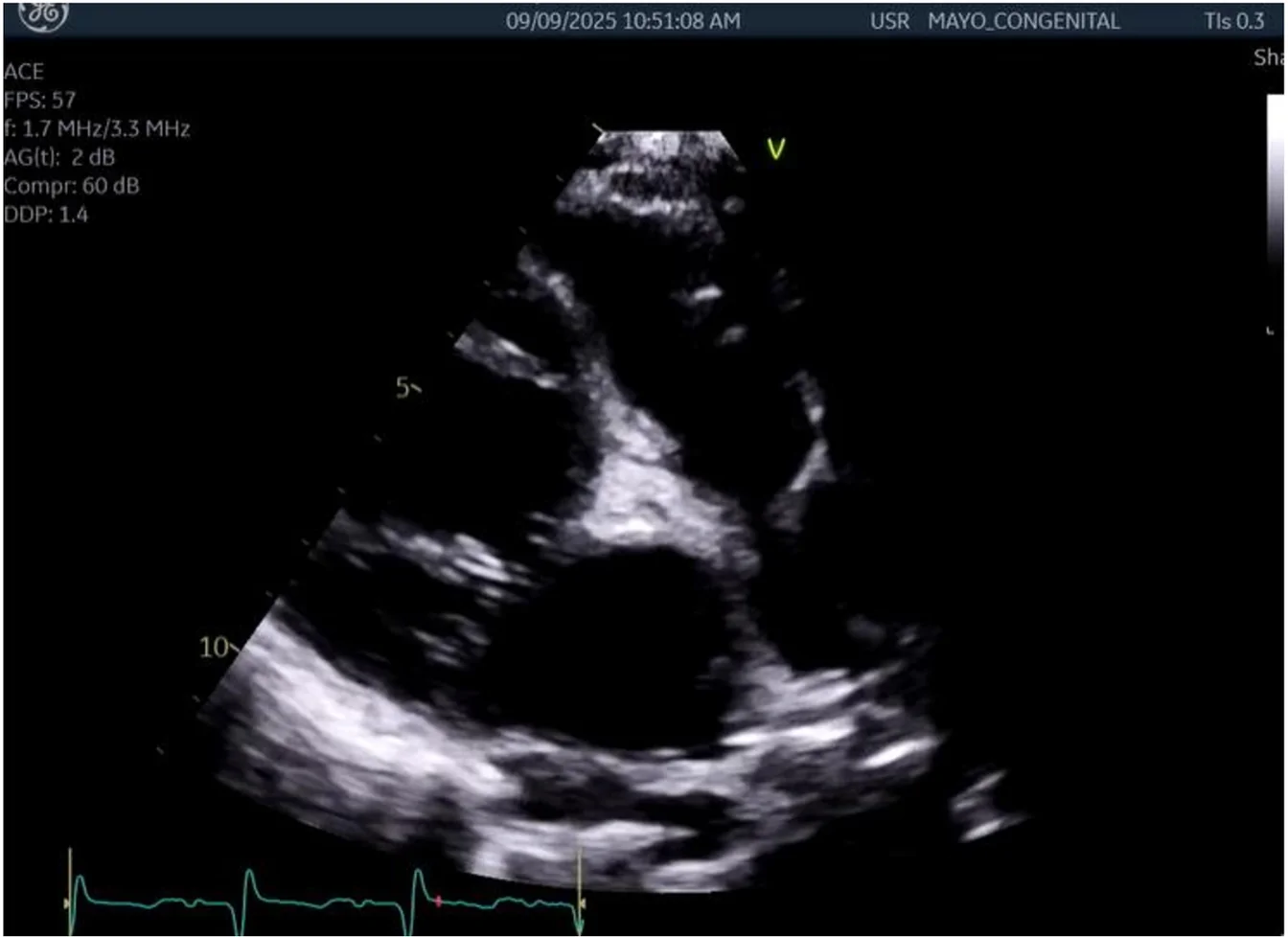
Transthoracic two-dimensional echocardiogram, parasternal long-axis view, demonstrating left ventricular chamber dilation consistent with dilated cardiomyopathy.

**Figure 2:**
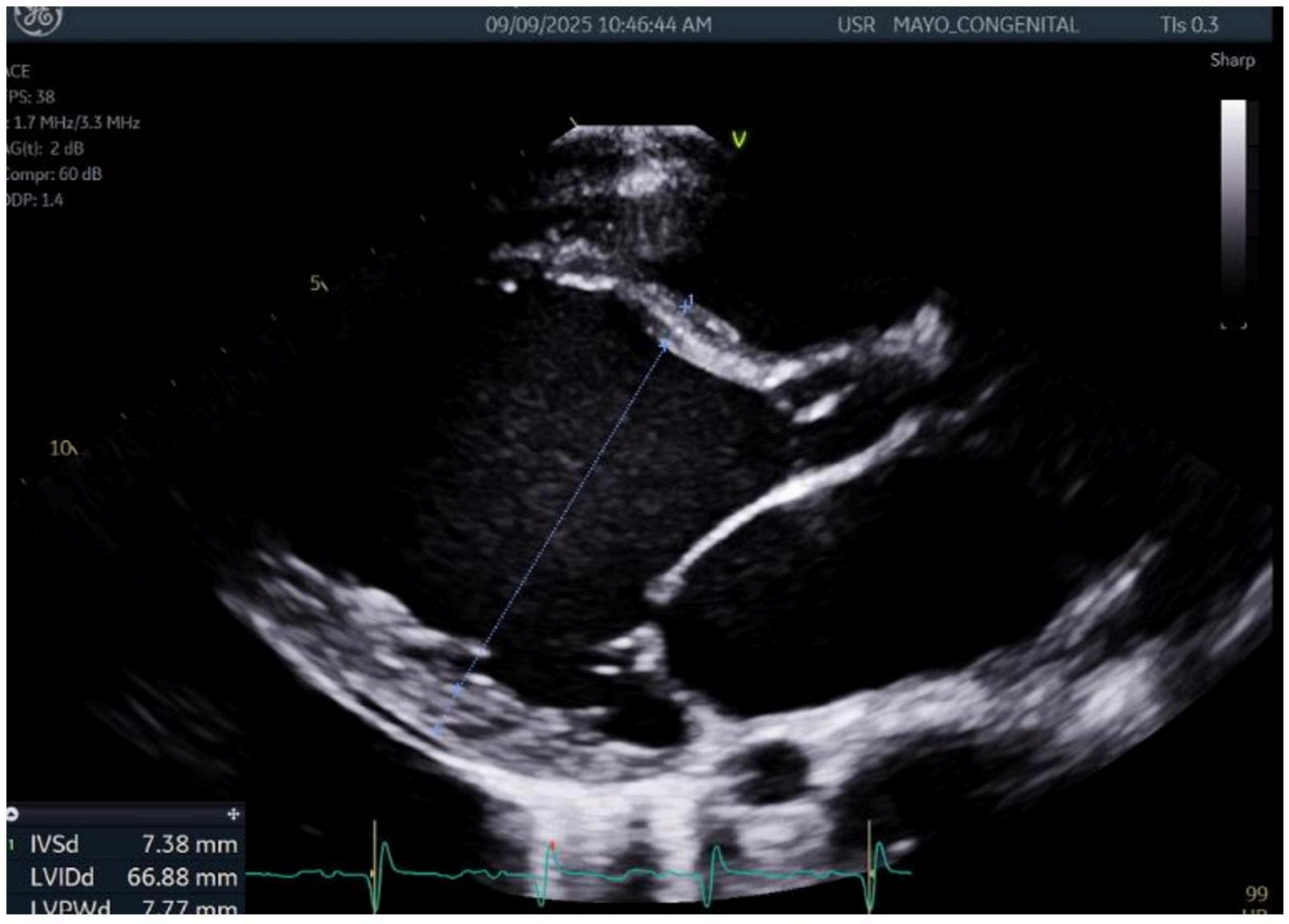
Transthoracic two-dimensional echocardiogram, parasternal long-axis view, with linear chamber quantification demonstrating a dilated left ventricle (LV internal diameter in diastole 66.9 mm) with normal wall thickness (interventricular septum 7.4 mm; posterior wall 7.8 mm), confirming the eccentric remodeling pattern of dilated cardiomyopathy.

Her clinical course was complicated by multiple episodes of ventricular tachycardia, unrelated to electrolyte abnormalities, with progressive deterioration, prompting transfer to the Cardiovascular ICU. Vasoactive medications, including intravenous milrinone and epinephrine infusions, along with intravenous calcium chloride, were administered. Mechanical circulatory support as a bridge to cardiac transplantation was discussed extensively with the family. Given their Amish background, extensive discussions with their community resulted in the family declining to pursue cardiac transplantation and redirecting their focus on comfort care measures. She was discharged home on hospice status per family preference prior to receiving the results of the metabolic workup.

Subsequently, metabolic workup revealed an abnormal plasma acylcarnitine profile with markedly elevated propionylcarnitine (C3) without evidence of secondary carnitine deficiency. Urine organic acid analysis demonstrated elevated excretion of 2-methylcitric acid, tiglylcarnitine, and trace amounts of 3-hydroxypropionic acid, along with mild ketonuria (Supplementary Tables S1–S7). These findings were consistent with a biochemical diagnosis of variant propionic acidemia. Notably, the patient did not exhibit hyperammonemia, lactic acidosis, or hypoglycemia during hospitalization, suggesting a milder phenotype.

She was subsequently readmitted to initiate treatment with IV carnitine after results of the metabolic workup became available. She received IV carnitine 100 mg/kg/dose every 6 h and an MMA/PA metabolic formula to assess clinical improvement, particularly cardiac function, with these interventions. Feeds were gradually advanced to goal as tolerated in the context of refeeding syndrome. At the time of readmission, the family had described a subjective improvement in her symptoms; however, this was not objectively evident based on her vital signs and clinical status. A repeat bedside echocardiogram following initiation of the specialized diet and IV carnitine demonstrated persistently reduced left ventricular systolic function, with an estimated ejection fraction of 15%, despite interventions.

In alignment with the family's goals of care, she was discharged home under hospice status. Genetic testing was recommended to confirm the genotype and facilitate cascade testing for first-degree relatives; however, it was not completed due to cost concerns (Figure 3).

**Figure 3:**
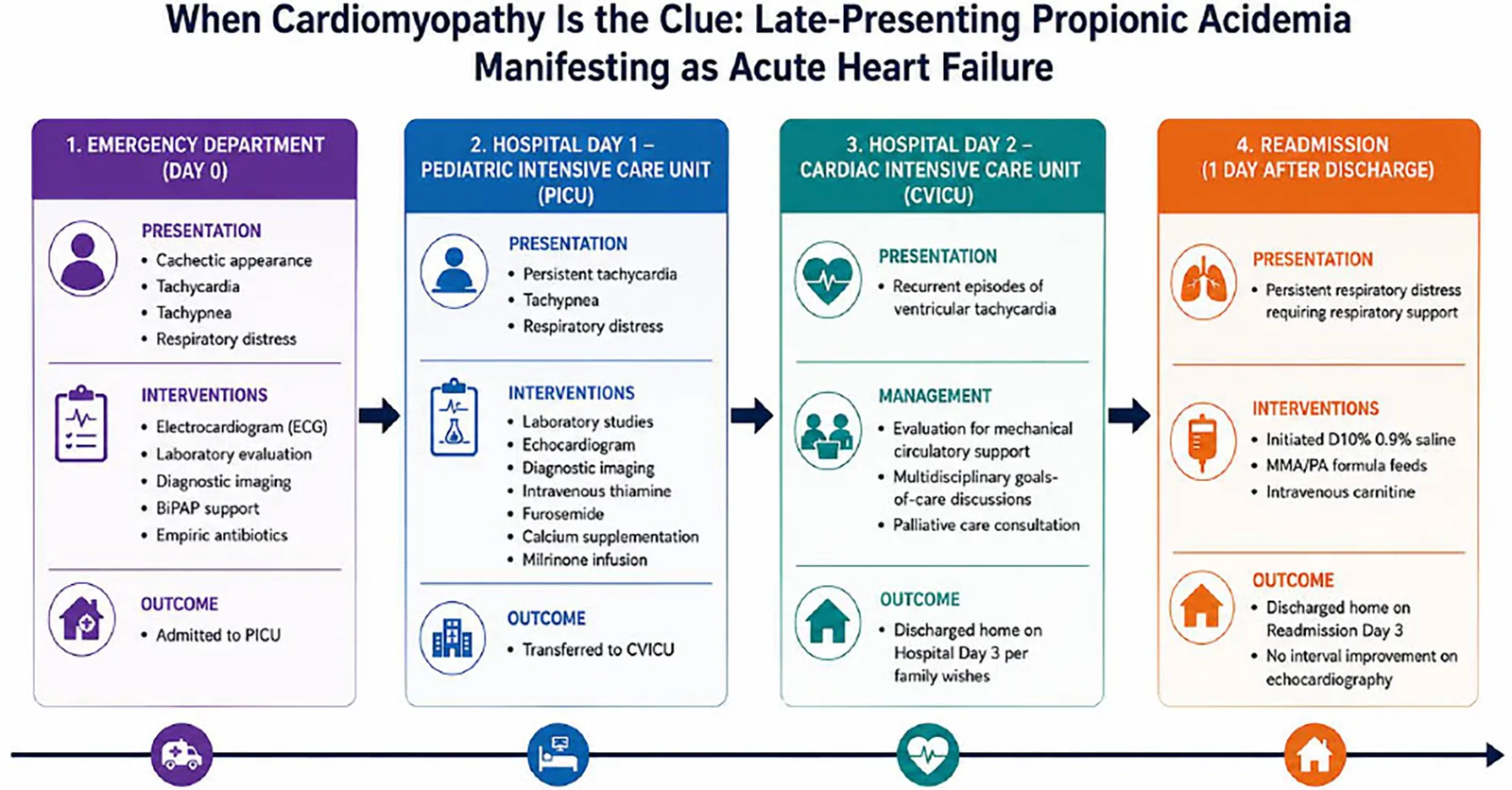
Timeline of the clinical course of a patient with late-presenting propionic acidemia manifesting as acute heart failure.

## Discussion

This case highlights important aspects of late-presenting propionic acidemia (PA) as acute decompensated heart failure secondary to dilated cardiomyopathy. Cardiomyopathy in propionic acidemia is multifactorial, primarily driven by accumulation of propionyl-CoA and its toxic metabolites (propionic acid, 3-hydroxypropionic acid, and methylcitric acid) due to deficient propionyl-CoA carboxylase (PCC) activity. This directly impairs the mitochondrial bioenergetics and calcium homeostasis resulting in chronic myocardial energy failure (4–6).

Reduced genetic diversity in Amish communities leads to an increased prevalence of inborn errors of metabolism like PA compared to the general population (7). While all states in the US include the disorder on newborn screening, many Amish patients do not undergo testing. It should be noted that normal or borderline newborn screen results do not necessarily rule out hypomorphic forms of this disease (8). The term variant propionic acidemia is used analogously to describe milder late-onset form of PA (9).

In the general population, PA arises from pathogenic variants in *PCCA* or *PCCB* (4, 19)*.* Late-onset propionic acidemia presenting primarily with dilated cardiomyopathy (DCM) is an increasingly recognized but likely underdiagnosed entity. A previously healthy adolescent of consanguineous origin was reported to present with acute DCM as the sole manifestation of PA, harboring a homozygous *PCCB* mutation (c.1229G > A; p.R410Q) with approximately 14% residual PCC activity, which likely explained the prolonged asymptomatic period (10). Another case involved a 17-year-old with late-onset PA who developed severe decompensated DCM with markedly decreased myocardial coenzyme Q10 levels (224 vs. 942–2,738 pmol/mg normal), providing the first direct evidence of secondary mitochondrial dysfunction as a pathophysiological mechanism (11). More recently, three Amish adolescents with a milder PA phenotype due to a PCCB founder variant (c.1606A > G; p.Asn536Asp) presented with isolated DCM and end-stage heart failure, with outcomes ranging from successful isolated heart transplantation to recurrent cardiogenic shock post-transplant due to metabolic decompensation, ultimately leading to combined heart-liver transplantation in a subsequent patient (12). Evidence suggests that liver transplantation improves metabolic stability, while heart transplantation, which is increasingly performed in combination with liver transplantation, is generally reserved for patients with end-stage cardiomyopathy (12, 13).

In the Amish, PA arises from a founder's homozygous missense variant in *PCCB* (c.1606A > G; p.Asn536Asp). These patients may exhibit some residual enzymatic activity in propionyl-coA carboxylase, which may result in false-negative newborn screening and contribute to clinical variability in disease course (14). In the general population, PA typically presents as a metabolic crisis in infancy. Published reports of Amish patients suggest a clinically variable, often milder phenotype. Chronically, they may exhibit developmental delays and poor growth without clinical decompensation until a later age, often secondary to an inciting event like viral or bacterial illness (10). In late-presenting patients, decompensated heart failure secondary to dilated cardiomyopathy may be the primary presenting symptom (15, 16). Invasive procedures like ECMO, as well as heart transplantation, are commonly deferred due to religious beliefs and the community's approach to communal financial resource stewardship (17).

Many of these important features were exhibited in our patient. She did not undergo newborn screening, exhibited cognitive delays and poor growth, had no cardiac history, and was otherwise considered healthy by her family. Shortly after a suspected viral illness, she presented acutely in her teenage years with acute decompensated heart failure secondary to dilated cardiomyopathy. Further investigation revealed an extensive maternal family history of similarly affected family members with heart failure due to PA. Transplantation was discussed and declined by the family after further discussion with their community leaders.

A notable limitation of this case is the absence of molecular genetic confirmation, as the family declined genetic testing due to cost concerns. GeneReviews states that definitive confirmation of PA relies on detection of biallelic pathogenic variants in PCCA or PCCB by molecular genetic testing or detection of deficient PCC enzymatic activity (18). With the combination of a positive family history of cardiomyopathy and a biochemical profile consistent with PA—including elevated propionylcarnitine (C3) and increased urinary 2-methylcitric acid—the diagnosis in this patient can be reasonably inferred as PA rather than other inherited causes of dilated cardiomyopathy. However, the limitation imposed by the absence of molecular confirmation is acknowledged. It should also be considered that PA may have contributed to, rather than solely caused, the cardiomyopathy in this patient. Dilated cardiomyopathy is an extremely heterogeneous condition (2, 15). Clinicians in states with large Amish populations should consider inborn errors of metabolism like PA in patients presenting with acute decompensated heart failure and no prior history of cardiac disease. Even in patients with normal newborn screen results, the disorder cannot be ruled out. Further history taking may reveal cognitive delays, poor growth, and an extensive family history of poorly defined heart failure. Genetic counseling should be incorporated into the diagnostic evaluation to facilitate discussion of inheritance patterns, implications for affected and at-risk family members, and reproductive considerations. Care should be taken to involve family members as well as faith and community leaders into care discussions, especially those involving invasive procedures and organ transplantation.

## Data Availability

The original contributions presented in the study are included in the article/Supplementary Material, further inquiries can be directed to the corresponding author.
