# Supplementary material for: Case Report: When cardiomyopathy is the clue: late-presenting propionic acidemia manifesting as acute heart failure
Source: Front Pediatr. 2026 Oct 5;14:1891137. doi: 10.3389/fped.2026.1891137 (PMC13677932; doi:10.3389/fped.2026.1891137)
Supplement: Supplementary file 1 [file Datasheet1.pdf]

CARE case report guidelines

CARE Checklist of information to include when writing a case report

(cc) BY-NC-ND

Topic	Item	Checklist item description	Reported on Line
**Title**	1	The diagnosis or intervention of primary focus followed by the words "case report"	Title
**Key Words**	2	2 to 5 key words that identify diagnoses or interventions in this case report, including "case report"	Keywords
**Abstract (no references)**	3a	Introduction: What is unique about this case and what does it add to the scientific literature?	Abstract
	3b	Main symptoms and/or important clinical findings	Abstract
	3c	The main diagnoses, therapeutic interventions, and outcomes	Abstract
	3d	Conclusion—What is the main "take-away" lesson(s) from this case?	Abstract
**Introduction**	4	One or two paragraphs summarizing why this case is unique (**may include references**)	Introduction
**Patient Information**	5a	De-identified patient specific information.	Case Presentation
	5b	Primary concerns and symptoms of the patient.	Case Presentation
	5c	Medical, family, and psycho-social history including relevant genetic information	Case Presentation
	5d	Relevant past interventions with outcomes	Case Presentation
**Clinical Findings**	6	Describe significant physical examination (PE) and important clinical findings.	Case Presentation
**Timeline**	7	Historical and current information from this episode of care organized as a timeline	Case Presentation
**Diagnostic Assessment**	8a	Diagnostic testing (such as PE, laboratory testing, imaging, surveys).	Case Presentation
	8b	Diagnostic challenges (such as access to testing, financial, or cultural)	Case Presentation
	8c	Diagnosis (including other diagnoses considered)	Case Presentation
	8d	Prognosis (such as staging in oncology) where applicable	Case Presentation
**Therapeutic Intervention**	9a	Types of therapeutic intervention (such as pharmacologic, surgical, preventive, self-care)	Case Presentation
	9b	Administration of therapeutic intervention (such as dosage, strength, duration)	Case Presentation
	9c	Changes in therapeutic intervention (with rationale)	Case Presentation
**Follow-up and Outcomes**	10a	Clinician and patient-assessed outcomes (if available)	Case Presentation
	10b	Important follow-up diagnostic and other test results	Case Presentation
	10c	Intervention adherence and tolerability (How was this assessed?)	Case Presentation
	10d	Adverse and unanticipated events	Case Presentation
**Discussion**	11a	A scientific discussion of the strengths AND limitations associated with this case report	Discussion
	11b	Discussion of the relevant medical literature **with references**.	Discussion
	11c	The scientific rationale for any conclusions (including assessment of possible causes)	Discussion
	11d	The primary "take-away" lessons of this case report (without references) in a one paragraph conclusion	Conclusion
**Patient Perspective**	12	The patient should share their perspective in one to two paragraphs on the treatment(s) they received	Case Presentation
**Informed Consent**	13	Did the patient give informed consent? Please provide if requested Ethics Statement	Yes ☒ No ☐
